# Physiological Condition of Juvenile Wading Birds in Relation to Multiple Landscape Stressors in the Florida Everglades: Effects of Hydrology, Prey Availability, and Mercury Bioaccumulation

**DOI:** 10.1371/journal.pone.0106447

**Published:** 2014-09-03

**Authors:** Garth Herring, Collin A. Eagles-Smith, Dale E. Gawlik, James M. Beerens, Joshua T. Ackerman

**Affiliations:** 1 United States Geological Survey, Forest and Rangeland Ecosystem Science Center, Corvallis, Oregon, United States of America; 2 Department of Biological Sciences, Florida Atlantic University, Boca Raton, Florida, United States of America; 3 United States Geological Survey, Western Ecological Research Center, Dixon Field Station, Dixon, California, United States of America; Central South University, China

## Abstract

The physiological condition of juvenile birds can be influenced by multiple ecological stressors, and few studies have concurrently considered the effects of environmental contaminants in combination with ecological attributes that can influence foraging conditions and prey availability. Using three temporally distinct indices of physiological condition, we compared the physiological response of nestling great egrets (*Ardea alba*) and white ibises (*Eudocimus albus*) to changing prey availability, hydrology (water depth, recession rate), and mercury exposure in the Florida Everglades. We found that the physiological response of chicks varied between species and among environmental variables. Chick body condition (short-term index) and fecal corticosterone levels (medium-term) were influenced by wetland water depth, prey availability, region, and age, but not by mercury contamination. However, mercury exposure did influence heat shock protein 70 (HSP70) in egret chicks, indicating a longer-term physiological response to contamination. Our results indicate that the physiological condition of egret and ibis chicks were influenced by several environmental stressors, and the time frame of the effect may depend on the specialized foraging behavior of the adults provisioning the chicks.

## Introduction

Environmental stressors, such as contaminants, prey availability, and adverse weather, can elicit strong physiological responses from wild birds in order to help them overcome short-term challenges. These responses can be particularly harmful during the early stages of life if the response exceeds physiological norms [1]–[3]. Physiological [4] and behavioral [5] responses can manifest throughout the annual cycle by many avian species in order to cope with seasonal environmental stochasticity. Understanding avian responses to multiple environmental stressors, when one of those stressors is an environmental contaminant, is poorly understood, yet this situation is prevalent in many anthropogenically-impacted environments [6], [7].

Mercury (Hg) contamination and subsequent bioaccumulation in waterbirds is problematic in wetlands throughout the world. Inorganic Hg is converted into the toxic and bioaccumulative form methylmercury (MeHg) under biogeochemical conditions that are common in wetland ecosystems [6], [7]. Once methylated, MeHg biomagnifies as it is transferred through the food chain [8]–[10]. Environmentally relevant Hg concentrations in birds have been associated with a suite of impaired physiological and reproductive responses, including altered reproductive hormone levels [11], [12], compromised hypothalamic-pituitary-adrenal axis [13]–[15], and reduced reproductive performance [16].

The decline of several species of Everglades' wading bird between the 1930s and 2001 has been related to changes in prey availability and alterations to the ecosystem's hydrology [17]–[19] and possibly due to Hg exposure [20], [21]. Great egrets (*Ardea alba*; hereafter egret) and white ibis (*Eudocimus albus*; hereafter ibis) are two of the most common breeding waterbirds in the Everglades, and they exhibit different foraging strategies that influence their exposure to ecological stressors. Ibis are more limited in their use of habitats and available prey than egrets [22]. Specifically, Ibis select high-quality foraging patches that they then abandon relatively quickly once prey availability drops, whereas egrets tend to remain at foraging sites even when prey densities are low [22]. Although both species select different foraging habitats [23] nest survival for both species is similarly influenced by hydrological conditions and prey availability [24]. Furthermore, Hg concentrations differ between egrets and ibises in the Florida Everglades [10], [25]–[28], which is likely a function of their foraging ecology and prey selection [28].

Herein, we assessed the physiological response of juvenile egrets and ibis to changing prey availability, hydrology (water depth, recession rate), and mercury exposure. We measured physiological biomarkers and body condition for egret and ibis nestlings in two consecutive years that differed greatly in hydrologic conditions, prey availability, and Hg exposure. Additionally, we measured physiological biomarkers of environmental stressors that manifest across a range of different time frames. In particular, we used measures of physiological condition that we expected would represent a temporal continuum of responses relative to the age of wading bird chicks in our study, including a body-condition index (short-time frame; 1–2 days; [29]), fecal corticosterone metabolites [FCORT] (medium-time frame; 2–7 days; [30], [31]), and heat shock proteins 60 and 70 [HSP60, HSP70] (long-time frame: weeks - >1 month; [28], [32]–[34]). Corticosterone (CORT) is a hormone that serves as a signal to modify both behavior and metabolism during a period of acute stress. Corticosterone is released by the hypothalamo-pituitary-adrenal axis into the blood stream when birds experience acute stress, facilitating a rapid response to overcome the stressor [2], [35]. Variation in CORT levels in waterbirds have been correlated with a variety of factors including prey availability [31], [36], Hg exposure [12], [15], and changing hydrological conditions [37]. In contrast, heat shock proteins are highly conserved molecular chaperones that function to maintain optimal cell function and homeostasis [38], [39] by being amplified through up-regulation during periods of stress to minimize cell protein damage [40], [41]. Elevated heat shock proteins are commonly associated with responses to damage associated with heavy metals including Hg [42], [43], decreased food availability [31], and rapid changes in hydrological conditions at foraging sites for birds [37]. In order to better understand the potential conservation implications of exposure to multiple environmental stressors we simultaneously tested those effects on these physiological biomarkers and chick body condition.

## Methods

### Ethics Statement

We made every attempt to reduce disturbance, stress, and other impacts to target and non-target species during the course of this research. Florida Atlantic University Institutional Animal Care and Use Committee (Protocol A0534) approved this study, and we conducted the research under U.S. Geological Survey banding permit 23354 and Florida Fish and Wildlife Conservation Commission Scientific Research Permit WX04487.

### Study Area

In 2006 and 2007, we monitored egret and ibis nestlings in Florida Everglades wading bird colonies at the Arthur R. Marshall Loxahatchee National Wildlife Refuge (Lox; *n* = 8 colonies) in Palm Beach County, Water Conservation Area (WCA) 2A (*n* = 2 colonies) in Broward County, and WCA 3A (*n* = 5 colonies) in Broward and Miami-Dade Counties, Florida, USA (Figure S1). Colonies were located by using a combination of ground and aerial surveys [24], by tracking radio-tagged adults [23], and by visiting known colony sites (e.g., South Florida Wading Bird Reports, South Florida Water Management District). Although we attempted to sample colonies in which both species nested, only one species nested in five of the twelve colonies.

### Chick sampling

We surveyed nests within egret and ibis colonies that were associated with our radio-tagged adults ([23], [24], Figure S1). We revisited nests and resampled chicks (n = 61 egret and 65 ibis nests) approximately every 7 days, and determined the hatching order and post-hatch age of chicks.

We used FCORT as an index of stress because this approach minimizes handling-induced bias that results during bird sampling. We collected fecal material as described in [37], and fecal samples were stored in labeled cryovial tubes and placed on ice. We recorded mass for both species to the nearest 5 g using a spring scale and recorded all other morphometric measurements to the nearest 1 mm. We sampled all nestlings during the morning to minimize heat stress and time of day effects on physiological metrics.

We sampled blood from chicks to measure both HSPs and Hg concentrations. From each chick, we collected up to 2 ml of blood from the brachial vein using a 27-gauge needle. Blood samples were stored on ice in heparinized cryovials until transport to the laboratory. Following The Ornithological Council guidelines [44], we limited the total blood volume of blood collection to <1% of bird mass and thus had limited blood volume available for determining physiological parameters and Hg concentrations. Therefore, we separated plasma and RBC fractions in the laboratory via centrifugation (15 min, 5,000 rpm) and determined HSPs and THg concentrations in RBCs. Each fraction was stored frozen at −20°C until analyses could be determined.

### Laboratory Methods

Fecal samples were homogenized in the laboratory, divided into 2 equal 1-ml wet portions, and dried using a Labconco CentriVap Concentro (Labconco, Kansas City, MO). Dried samples (∼0.25 g) were then mixed with 5 ml of 95% ethanol and vortexed for 30 min. After centrifugation (15 min, 2500 × g) the supernatant was transferred to a new vial, and evaporated under a stream of nitrogen gas. Corticosterone metabolites were then resuspended in diluted extraction buffer and measured using the Correlate-EIA Corticosterone Enzyme Immunoassay Kit (EIA [45];) following the manufacturer's instructions (Assay Design, Inc., Ann Arbor, MI). Inter- and intra-assay coefficients of variation for FCORT internal standards were 7% and 11%, and 8% and 9% respectively for egrets and ibises. We validated that FCORT levels did not change after freezing as in the case of mammals [46] by freezing and measuring FCORT levels monthly for 6 months [45].

A subset of RBCs was washed three times using phosphate buffered saline, centrifuged and the pellet was removed after the final wash. The RBC pellet was then mixed with 1× extraction reagent and a protease inhibitor cocktail (Sigma), vortexed for 5 min and then sonicated for 1 min. Samples were again centrifuged (15 min, 2500 g) and the supernatant removed. We measured HSP60 (HSPD1) and HSP70 (HSP72/HSPA1A) in the supernatant using EIA kits specific to just those stress proteins and not all other HSP60 and HSP70 family members EIA kits (Assay Designs, Inc., Ann Arbor, MI). Inter- and intra-assay coefficients of variation for HSP60 and HSP70 internal standards were 5% and 7% and 6% and 7% respectively for egrets and ibis. All samples were run in duplicate, and means of duplicates were used in all analyses. All EIA kits were validated using serial dilutions and spike tests to determine percent recovery [45].

### Mercury Determination

We analyzed the remaining portion of RBCs for total mercury (THg) concentrations because the majority of Hg in blood is in the RBC fraction [47], [48] and RBC Hg concentrations are correlated with whole blood Hg concentrations [49], [50]. Further, almost all (>95%) of the mercury in avian blood is in the methylmercury form and THg concentrations are highly correlated with methylmercury levels [51], [52]. To minimize any potential variability in THg concentrations associated with processing and differential moisture loss during long-term storage of samples, RBC fractions were dried (48 hours at 50°C) to a constant weight before THg analysis. We measured up to 0.05 g of dried blood into nickel sample vessels (weighed to the nearest 0.00001 g; Mettler Toledo model XS105, Mettler Toledo, Columbus, Ohio, USA). Following U.S. Environmental Protection Agency method 7473 [53], we determined THg concentrations in each RBC sample on a Milestone DMA-80 direct mercury analyzer (Milestone, Monroe, Connecticut, USA). Analytical equipment was calibrated using certified standard solutions prior to analysis, and accuracy and precision were evaluated within each analytical batch through the inclusion of certified reference materials (either dogfish muscle tissue [DORM-3] or dogfish liver [DOLT-4] by the National Research Council of Canada, Ottawa, Canada), calibration verifications (liquid standards), duplicates, and blanks. Recoveries averaged 102.4±0.06% (*n = *17) and 97.7±0.04% (*n = *33) for certified reference materials and calibration checks, respectively. Absolute relative percent difference for all duplicates averaged 1.7%±0.08% (*n* = 16).

### Hydrological Conditions

We used the Everglades Depth Estimation Network (EDEN [54];) to estimate water depths and water level recession rates within foraging ranges of adults associated with breeding colonies where chicks were sampled. The EDEN uses a system of water level gauges to produce a water surface model coupled with a ground elevation model to estimate water depth for the entire freshwater portion of the Greater Everglades [55]. The EDEN calculates water level depths in 400 m×400 m grid cells at daily time steps accounting for evapotranspiration, rainfall, and sheet flow. The estimated water depths have been found to be accurate to within 5 cm [55].

To estimate recession rates and water depths, we used protocols developed during previous studies [23], [24], [28]. Briefly we used fixed radius buffers (hereafter foraging range) associated with breeding colonies based on mean distances flown plus one standard error by radio tagged adult egrets and ibises in a concurrent study. We defined foraging range as the average distance that radio tagged adults flew from breeding colonies to a foraging site throughout the breeding season. We extracted the water depth from each EDEN grid cell within the foraging ranges for each specific colony, year, and species combination. For each day that we sampled chicks, we calculated water recession rate and water depth as the mean value for the preceding 2-week period [24], [28]. Positive recession rates indicate decreasing water levels. Surface water could still be present when estimated cell depth <0 cm; however, the 5-cm error in EDEN depths suggests that a majority of the ground surface would be exposed in a given 400 m×400 m grid cell.

### Statistical procedures

We used linear mixed-effects models in JMP using maximum likelihood estimation (JMP 2001 [56];) to test the effects of environmental stressors (water depth, recession rate, prey availability, and Hg) on the physiological condition (chick body condition index, FCORT, HSP70, and HSP60) of egret and ibis chicks. We included age of chicks in models to account for variation in prey selection behavior as chicks aged [57]–[59]. The hatch order of chicks within the nest was included to account for competition for food within nests [24], [28]. Lastly, we included region because our previous research found differences in adult physiological condition among Everglades' regions [37]. We did not include a year effect because it was confounded with large differences in prey biomass observed between years. For egret chick models, we statistically nested the chick identification code within the nest identification code and added it as a random effect to the model to account for the non-independence of sampling chicks multiple times and different numbers of chicks within the same nest. For ibis chick models, we included the nest identification code as a random effect to account for the non-independence of sampling multiple chicks within a nest. We used the maximum likelihood estimation method in our linear models. Because we intentionally studied two species with contrasting foraging strategies, we ran separate models for each species. Natural log transformations where used to improve normality of residuals for prey biomass, THg, FCORT, HSP60, and HSP70 data. We assessed the level of multicollinearity using the variance inflation factor (VIF; 60); across all models the VIFs ranged from 1.04–2.41 suggesting no significant multicollinearity. We calculated a marginal and conditional coefficient of determination (*R* ^2^) for each model to examine goodness-of-fit in R [GLMM *R* ^2^; 61]. Briefly, for mixed effects models, the marginal *R* ^2^ (*R* ^2^_GLMM(m)_) reports the goodness-of-fit for just the fixed effects, while the conditional *R* ^2^ (*R* ^2^_GLMM(c)_) incorporates both the random and fixed effects as a measure of explained variation [61].

In the case of chick mass, we did not have repeated measurements of all chicks across time and subsequently could not calculate growth rates. Instead, we calculated a chick body condition index that allowed us to examine the potential effects of multiple landscape stressors on a relative measure of the condition of the chick that was independent of the mass of a chick [29], [62]. To do so, we first we calculated a structural body size index using principal components analysis on the morphometric measurements (e.g. tarsus, bill length, wing length) for each species separately. We then regressed chick mass on tarsus length (the variable our principal components analysis identified as the best predictor of structural size for both egrets and ibis), including the first principal component as a covariate. We then used the residuals expressed as a percentage of the predicted mass, to obtain our chick body condition index [29], [62]. Benson *et al.* [29] found that measurements taken during a single visit to a colony were useful to detect variation in chick body condition across time.

## Results

During the 2006 and 2007 nesting seasons we collected blood samples from 254 chicks (169 egret; 2006 = 27, 2007 = 142, and 85 ibis; 2006 = 30, 2007 = 55). The geometric mean across all dates, regions, and hatch order was 6.99±0.63 (SE) in egrets and 4.57±0.83 in ibises for FCORT concentration (µg/g), 13.73±0.96 in egrets and 14.73±2.06 in ibis for HSP60 concentration (ng/ml), 4.44±0.44 in egrets and 2.53±0.35 in ibis for HSP70 concentration (ng/ml), and 4.13±0.16 in egrets and 0.58±0.07 in ibis for THg concentration (µg/g dw).

### Chick body condition index

**Figure 1:**
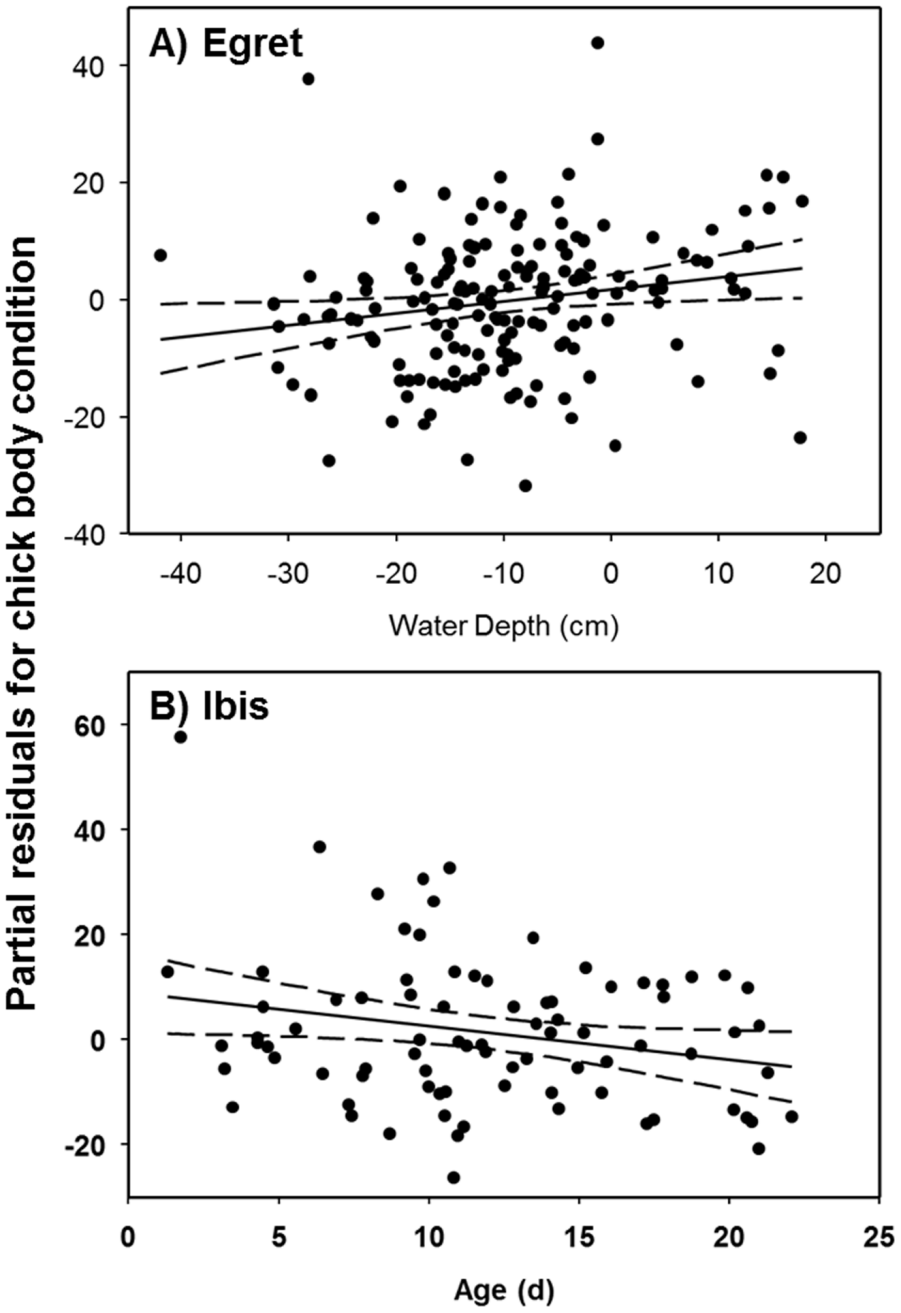
Relationship between partial residuals of chick body condition index and water depth (A; cm/^2^) for great egret chicks and age (B; days) for white ibis chicks in the Florida Everglades, after accounting for other model variables. Dashed lines indicated 95% confidence.

Egret chick body condition was influenced by water depth (*F*_1, 128.8_ = 6.60, *P* = 0.01), but not region (*F*_2, 114.7_ = 2.30, *P* = 0.10), water recession rate (*F*_1, 119.2_ = 2.28, *P* = 0.14), prey biomass (*F*_1, 142.7_ = 0.38, *P* = 0.53), age (*F*_1, 52.4_ = 0.43, *P* = 0.51), hatch order (*F*_1, 79.9_ = 1.95, *P* = 0.17), nor Hg concentration (*F*_1, 150.8_ = 0.02, *P* = 0.88). The low marginal *R* ^2^ value of our egret chick body condition model (*R* ^2^_GLMM(m)_ = 0.10, *R* ^2^_GLMM(c)_ = 0.21) suggests that the model was a relatively poor predictor of body condition. However, egret chick body condition increased with increasing water depth (*β* = 0.20±0.13–95% confidence interval) on average by 23% across the range of water depths observed (range = −46 to 17 cm; Figure 1A).

Conversely, ibis chick body condition was influenced by both region (*F*_1, 37.6_ = 9.09, *P* = 0.004) and age (*F*_1, 75_ = 5.26, *P* = 0.02), but not by water depth (*F*_1, 68.8_ = 0.0001, *P* = 0.99), water recession rate (*F*_1, 72.5_ = 0.005, *P* = 0.98), prey biomass (*F*_1, 55.6_ = 0.06, *P* = 0.80), hatch order (*F*_1, 38.6_ = 33, *P* = 0.56), nor Hg concentration (*F*_1, 75_ = 0.10, *P* = 0.75). The ibis chick body condition model marginal *R* ^2^ value (*R* ^2^_GLMM(m)_ = 0.23), suggested that the model was a modest predictor of ibis chick body condition. Similarity between the marginal *R* ^2^ value and the conditional *R* ^2^ (*R* ^2^_GLMM(c)_ = 0.23) suggested that little additional variance was explained by the random effect associated with sampling multiple chicks within nests. Ibis chick body condition was 1.9 times higher in Lox (mean = 5.36±2.68 SE) than WCA3A Lox (−10.02±4.31 SE; *β* = 7.69±4.99) and decreased by 26% across the range of ages (*β* = −0.64±0.54, range = 3–30 days; Figure 1B).

### Fecal corticosterone metabolites

**Figure 2:**
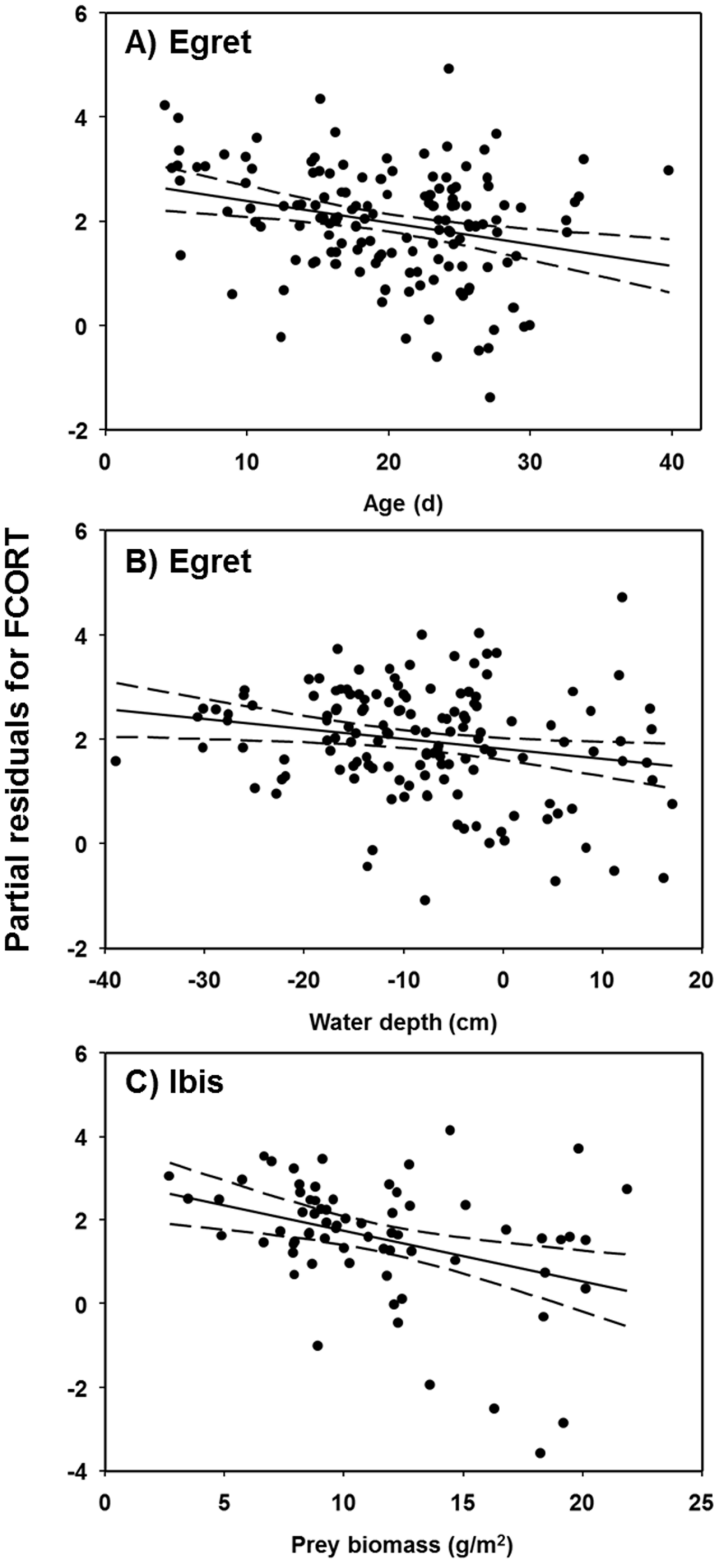
Relationship between partial residuals of fecal corticosterone metabolite (µg/g dw) and chick age (A; days) and water depth (B; cm) for great egret chicks and prey biomass (C; g/m^2^) for white ibis in the Florida Everglades, after accounting for other model variables. Dashed lines indicated 95% confidence interval.

Egret FCORT metabolite concentrations were influenced by age (*F*_1, 13.5_ = 13.50, *P*<0.001), water depth (*F*_1, 7.5_ = 7.50, *P* = 0.007), and region (*F*_1, 108.6_ = 3.06, *P* = 0.007), but not by water recession rate (*F*_1, 106.8_ = 0.01, *P* = 0.97), prey biomass (*F*_1, 124.9_ = 2.82, *P* = 0.09), hatch order (*F*_1, 80.5_ = 0.78, *P* = 0.38), nor Hg concentration (*F*_1, 133.6_ = 1.65, *P* = 0.20). The similarity between the marginal and conditional *R* ^2^ values (*R* ^2^_GLMM(m)_ = 0.34, *R* ^2^_GLMM(c)_ = 0.37) suggests that the fixed effects alone were considerably more influential than the random effects associated with sampling multiple chicks within the same nest on more than one occasion. Egret FCORT metabolite concentrations decreased with increasing age (*β* = −0.04±0.02) and water depth (*β* = −0.02±0.01) on average by 79% across the range of ages (range = 3–42; Figure 2A) and 72% across the range of water depths (range = −46 to 17 cm; Figure 2B). Egret FCORT metabolite concentrations were higher in WCA3A (11.74±2.45 SE) than either WCA2A (*t*_118.3_ = 2.09, *P* = 0.04; mean = 4.71±1.60 SE) or Lox (*t*_102.3_ = 2.25, *P* = 0.03; mean  = 6.34±0.76 SE) respectively, but similar between WCA2A and Lox (*t*_114.5_ = −0.83, *P* = 0.40).

Similar to egrets, region influenced ibis FCORT metabolite concentrations (*F*_1, 54.8_ = 4.40, *P* = 0.04), however unlike egrets, prey biomass also influenced ibis FCORT metabolites (*F*_1, 58.4_ = 19.41, *P*<0.001). Ibis FCORT metabolites were not influenced by water depth (*F*_1, 59.6_ = 0.65, *P* = 0.42), water recession rate (*F*_1, 60.4_ = 0.36, *P* = 0.55), age (*F*_1, 60.8_ = 0.25, *P* = 0.62), hatch order (*F*_1, 46.1_ = 1.43, *P* = 0.23), nor Hg concentration (*F*_1, 59.1_ = 0.001, *P* = 0.96). The ibis FCORT model had a moderate marginal *R* ^2^ value (*R* ^2^_GLMM(m)_ = 0.36), but the conditional *R* ^2^ value (*R* ^2^_GLMM(c)_ = 0.71) suggested that a similar amount of the variability in ibis FCORT concentrations were associated with just the random effect of sampling multiple chicks within the same nest. FCORT metabolite concentrations were 3.3 times higher in Lox (mean = 5.26±1.68 SE) than WCA3A (1.60±0.70 SE; *Β* = 0.59±0.54), and decreased by 98% (*Β* = −1.40±0.62), across the range of prey biomass (range = 1.8–26.7 g/m^2^; Figure 2C).

### Heat shock protein 70

**Figure 3:**
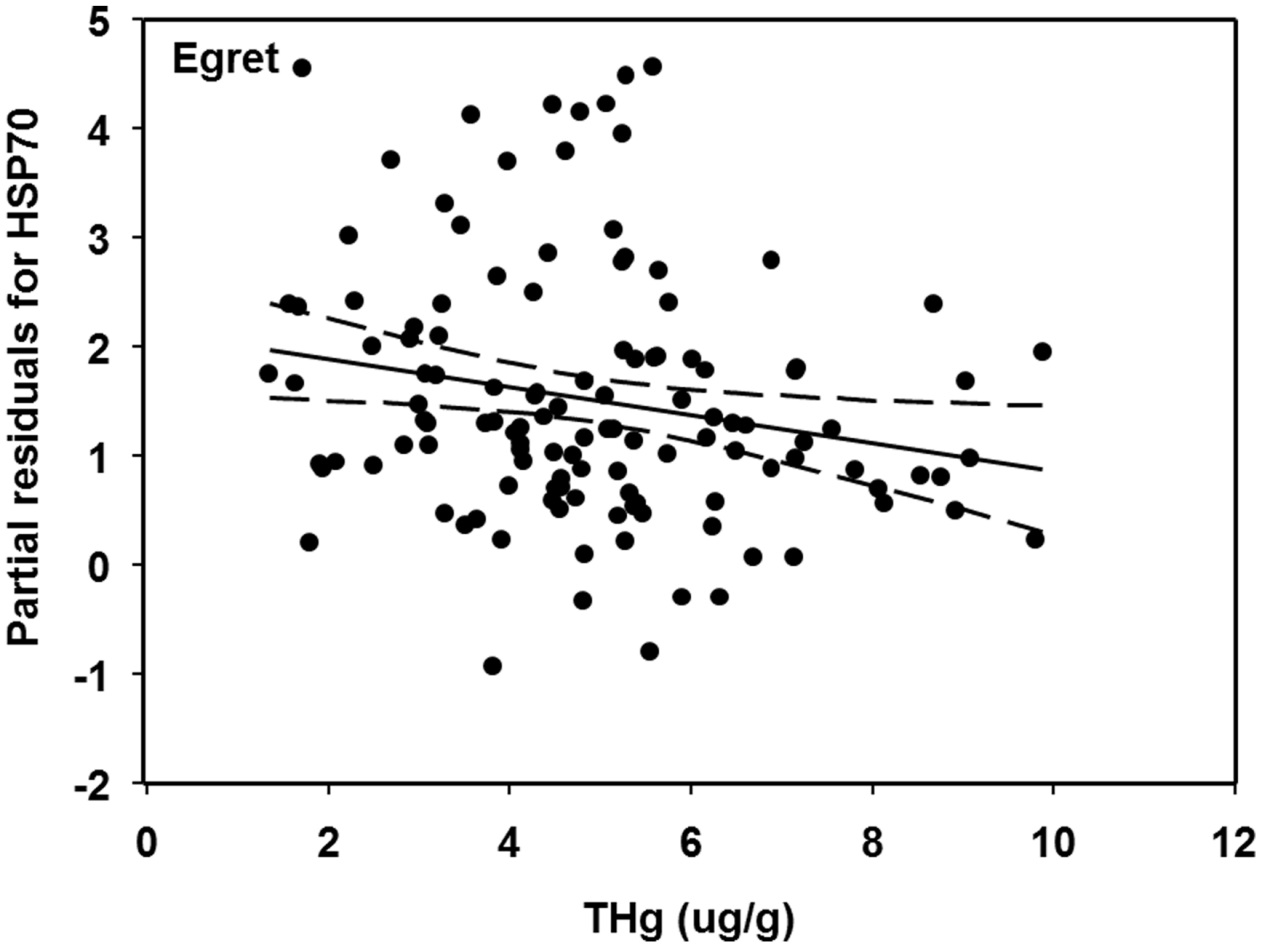
Partial residuals of HSP70 concentrations (ng/ml) and red blood cell THg (µg/g dw) for great egret chicks in the Florida Everglades, after accounting for other model variables. Dashed lines indicated 95% confidence interval.

Egret HSP70 concentrations were influenced by Hg concentration (*F*_1, 109.9_ = 4.09, *P* = 0.04), but not region (*F*_2, 100.3_ = 1.56, *P* = 0.21), water depth (*F*_1_, 101.2 = 0.16, *P* = 0.68), water recession rate (*F*_1, 91.0_ = 0.37, *P* = 0.54), prey biomass (*F*_1, 99.36_ = 0.23, *P* = 0.62), age (*F*_1, 110.8_ = 0.02, *P* = 0.87), nor hatch order (*F*_1, 76.6_ = 1.63, *P* = 0.20). The overall amount of variation in egret HSP70 explained by the fixed effects was relatively low (*R* ^2^_GLMM(m)_ = 0.13), however very little of that variation was explained by our random effects (*R* ^2^_GLMM(c)_ = 0.16) associated sampling multiple chicks from the same nest or sampling those chicks on several occasions. The beta coefficient estimate for Hg (*β* = −0.48±0.22) indicated a decrease in HSP70 concentrations of 83% across the range Hg values (range = 0.26–11.01 µg/g dw; Figure 3).

In the case of ibis, neither water depth (*F*_1, 30.9_ = 0.12, *P* = 0.72), region (*F*_1, 28.6_ = 0.11, *P* = 0.73), water recession rate (*F*_1, 32.9_ = 0.0009, *P* = 0.97), prey biomass (*F*_1, 32.2_ = 0.12, *P* = 0.72), age (*F*_1, 31.4_ = 0.49, *P* = 0.48), hatch order (*F*_1, 29.5_ = 0.02, *P* = 0.87), nor Hg concentration (*F*_1, 13.9_ = 0.0001, *P* = 0.99) influenced HSP70 concentrations. Correspondingly, the fixed effects (*R* ^2^_GLMM(m)_ = 0.06) explained very little of the variation in HSP70, however the conditional effects (*R* ^2^_GLMM(c)_ = 0.74) associated with sampling chicks from the same nest played a considerably larger role in ibis HSP70 concentrations.

### Heat shock protein 60

Egret chick HSP60 concentrations were not influenced by water depth (*F*_1, 118.9_ = 0.0002, *P* = 0.98), region (*F*_2, 111.4_ = 0.01, *P* = 0.98), water recession rate (*F*_1, 119.7_ = 0.27, *P* = 0.60), prey biomass (*F*_1, 86.6_ = 0.91, *P* = 0.34), age (*F*_1, 134.5_ = 1.81, *P* = 0.18), hatch order (*F*_1, 81.7_ = 0.05, *P* = 0.81), nor Hg concentration (*F*_1, 120.2_ = 0.86, *P* = 0.34). The low *R* ^2^ values associated with both the fixed and random effects suggested that variation in egret HSP60 results from factors other than those measured in this study (*R* ^2^_GLMM(m)_ = 0.04, *R* ^2^_GLMM(c)_ = 0.04).

**Figure 4:**
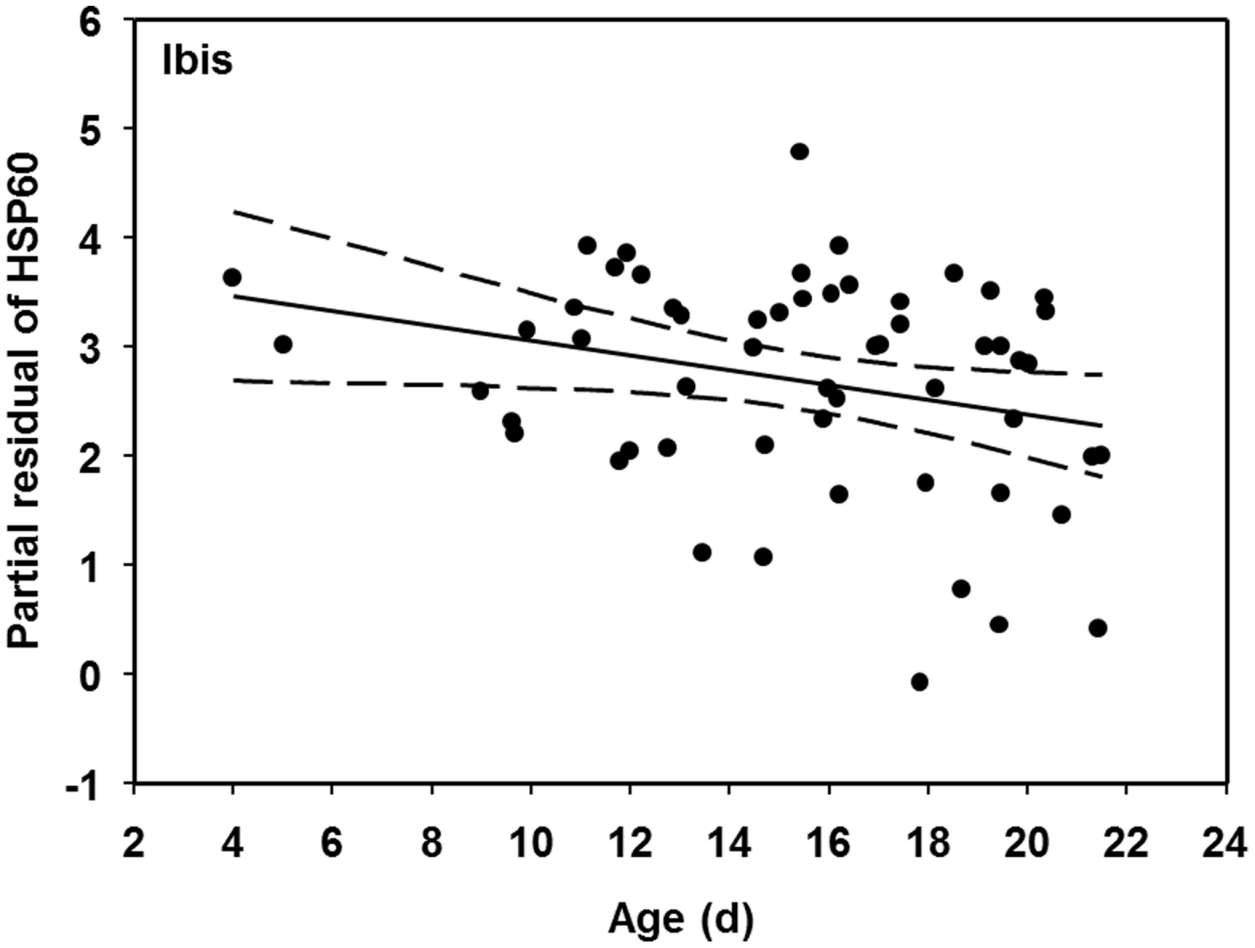
Relationship between partial residuals of HSP60 (ng/ml) and chick age (days) for white ibis chicks in the Florida Everglades, after accounting for other model variables. Dashed lines indicated 95% confidence interval.

In the case of Ibis HSP60 concentrations were influenced by age (*F*_1_, _43.4_ = 4.32, *P* = 0.04), but not prey biomass (*F*_1, 45.5_ = 0.89, *P* = 0.35), water depth (*F*_1, 42.0_ = 0.11, *P* = 0.74), water recession rate (*F*_1, 46.0_ = 0.04, *P* = 0.83), hatch order (*F*_1, 31.6_ = 0.02, *P* = 0.87), nor Hg concentration (*F*_1, 46.6_ = 1.61, *P* = 0.21). The overall amount of variation in ibis HSP60 explained by the fixed effects was relatively low (*R* ^2^_GLMM(m)_ = 0.15), but the random effect (*R* ^2^_GLMM(c)_ = 0.89) associated with sampling multiple chicks from the same nest explained considerably more of the variation in ibis HSP60 concentrations. The beta coefficient estimates for age (*β* = −0.05±0.04) indicated HSP concentrations declined by 74% across the range of ages (range = 3–30 days; Figure 4).

## Discussion

Different environmental stressors were associated with different time frames and biomarkers of physiological condition in egret and ibis chicks. The short and moderate time frame markers, chick body condition and FCORT, respectively, were correlated with landscape variables such as prey biomass and water depth, whereas there was no measurable effect of Hg. In contrast, the long-term physiological markers were not influenced by any of the measured landscape stressors, but one (HSP70 in egrets) was influenced by Hg concentration. One potential confounding aspect in our study is the fact that the same hydrology variables used to define the landscape stressors that we tested to determine their influence on physiological condition are also know to influence concentrations of Hg in egret and ibis adults and chicks [28]. Previously, Herring *et al.* [28] found that water depths and recession rates, while not the primary variables that influenced Hg concentrations, did play a secondary role in influencing Hg concentrations in egrets and ibis. Disentangling those effects is beyond the scope of this study, but illustrates the complexity of examining different environmental stressors on the physiological condition of birds.

Egret HSP70 decreased by 83% across the range (low-high) of Hg concentrations, suggesting that Hg may have down-regulated these heat shock proteins. Previous research on heat shock protein regulation in relation to both inorganic Hg and MeHg have found that heat shock protein regulation can be either inhibited (down-regulated) or induced (up-regulated) depending on the chemical form of Hg [63]. Up-regulation of heat shock proteins are associated with protective stress responses to minimize cell protein damage [40], [41], [64]. Methylmercury exposure in birds is associated with oxidative stress in liver, kidney, and brain tissue [65], and up-regulation of heat shock proteins has recently been identified as a potential biological defense mechanism against MeHg toxicity [66], [67]. However, it is unclear what the consequences are for down-regulation of heat shock proteins as we observed with egrets. Presumably, a reduction of heat shock proteins could result in decreased cell function and homeostatic imbalance, given that this is the primary role heat shock proteins when an individual is not in a stressful situation [38], [39]. Opposite response patterns of other stress biomarkers in relation to Hg exposure have been documented in other birds (e.g., [15]). Herring *et al.* [15] found that Hg exposure in Forster's tern (*Sterna forsteri*) chicks depressed FCORT concentrations by up to 81% via the downregulation of the HPA axis. These results suggest that the physiological stress response systems of birds are dynamic and that Hg exposure can negatively influence critical physiological processes.

Current Hg exposure levels of egrets and ibises throughout much of the Florida Everglades are low relative to historical reports [28], [68]. However, the fact that for egrets, one of their HSPs was influenced by Hg, demonstrates that the current Hg levels could still influence the physiology of this species. Moreover, Hg hotspots still exist within the Florida Everglades (e.g., interior portions of Everglades National Park not sampled in this study) where Hg levels in fish far exceed levels measured anywhere else within the ecosystem, and still pose a risk to wading birds [10], [21]. Ibis heat shock proteins were not influenced by Hg exposure in this study, which was likely a function of Hg exposure levels being approximately 7 times lower than those of egrets.

We found that ibis but not egret FCORT levels were influenced by prey biomass levels, and that they declined by 98% across low-to-high range of prey biomass at foraging sites. This is consistent with responses in pre-breeding adult birds [37], suggesting that this relationship may be consistent across life stages. Similarly, ibis nest success was also more sensitive to changes in prey biomass than that of egrets [24], demonstrating the importance of prey biomass to ibis. Egret FCORT metabolite concentrations also declined by 79% across the age range (young to old) of chicks. Other studies of CORT stress levels in chicks have found that concentrations often increase as chicks get older, in preparation for fledging [3], [69], [70]. However, there is some evidence that elevated CORT levels can result in decreased protein deposition in growing feathers [71], which may lead to poorer feather quality [72]. Increased baseline CORT levels can also impair growth rates and immune function [73]. Alternatively egret CORT levels may be higher in younger chicks to facilitate improved begging to adults [74], [75] and promote growth at a critical stage in the chicks' development since CORT allows the organism to mobilize needed energy reserves [76].

We found support for the influence of hydrological variables on egret physiology, in particular egret chick body condition increased with increasing water depth. Critical to understanding these responses is the fact that the range of water depths associated with our study did not exceed the observed foraging range and optimal depths for egrets [23] and generally would be considered to be shallow for egret foraging [22]. As such, the increase in chick body condition is likely driven by the fact that egrets prefer larger prey items [57] found in deeper depths relative to those very shallow sites where large prey die rapidly. We expect that body condition would have dropped had depths increased beyond the normal foraging range of egrets.

Neither water depths nor recession rates influenced ibis chick physiological condition in the current study. Although recession rates were found to influence the pre-breeding physiological condition of ibis previously [37], recession rates during the nestling stage may play a smaller role in chick physiological condition because water depths have largely reached optimal foraging depths for adults around breeding colonies [22], [23]. Thus, small variation in water depth and recession rate likely have little influence on accessibility of foraging sites or the availability of prey for ibis. However, if rainfall events occur, rapid negative recession rates that result in increased water depths may result in the loss of foraging site access and reduced prey availability. Although these conditions did not occur in the present study, they can have a substantial effect on nestlings [77] and would be expected to decrease chick physiological condition. Similarly, water depths were largely within the foraging range and were near optimal within the proximity of breeding colonies during this study. In fact, water depths associated with ibises were less variable than those observed in egrets (coefficient of variation  = 20% vs 56% respectively). Under conditions with deeper or more variable water depths, we expect that ibises may have an increased physiological response due to their dependence on high quality (shallow water with high prey densities) foraging sites.

We also found that chick age was an influential variable in explaining physiological responses, including ibis body condition and HSP60, and egret FCORT. The body condition of ibis chicks declined 26% from the youngest to the oldest chicks. Although it is unclear why chick body condition declined as they aged, there was not a concomitant increase in stress biomarkers such as FCORT or HSPs. In fact, HSP60 levels actually declined by 74% across the range of ibis chick ages. One previous study found that chickens (*Gallus gallus domesticus*) that were experimentally fed lower energy diets, resulting in lower body masses, also had lower levels of HSPs relative to those with higher energy diets and higher masses [78]. Ibis body condition may have declined as the chicks prepared to fledge, a period where many bird species reduce their body mass/condition [79], [80]. It is believed that this reduction in overall mass allows birds to change from plump immobile chicks to slender and proficient flying juveniles [80]–[82].

### Conservation and Management Implications

There were two primary findings of this study that have the potential to benefit conservation of wading bird species. First, we found support for chick physiology to be influenced by hydrology variables such as water depths. Water managers have the potential to regulate the hydrology throughout much of the Everglades through a system of canals, levees, and pumps, theoretically allowing them to adjust water depths for wading birds. This hydrology variable has also been previously linked to pre-breeding adult physiological condition [37], nesting success [24], and foraging site selection of egrets and ibises [23]. Optimal water depths for foraging wading birds differ by species [22], [23], foraging strategies (searchers and exploiters), and years depending on prey biomass conditions; however maintaining water depths between −22 and 21 cm has been suggested to provide suitable foraging conditions for both species and foraging groups [23].

We also found that current levels of Hg exposure in the Everglades may influence the expression of heat shock proteins in egrets. Understanding what the consequences are for these changes in heat shock protein concentrations may provide insight as to how Hg may have impacted Everglades' wading bird populations in the past. This understanding may be relevant to future patterns of wading bird nesting because there are portions of the Everglades that still contain high levels of Hg in prey species of wading birds [10].

## Supporting Information

Figure S1 **Location of study area indicating wading bird nesting colonies sampled during 2006 and 2007.** The primary Water Management Areas (WCAs), Arthur R. Marshall Loxahatchee National Wildlife Refuge (Lox), and Everglades National Park (ENP) are indicated.
(TIF)

Table S1 **Data used in analyses of the Physiological condition of juvenile wading birds in relation to multiple landscape stressors in the Florida Everglades: effects of hydrology, prey availability, and mercury bioaccumulation.**
(PDF)
