# Supplementary figures and images for: Physiological Condition of Juvenile Wading Birds in Relation to Multiple Landscape Stressors in the Florida Everglades: Effects of Hydrology, Prey Availability, and Mercury Bioaccumulation
Source: PLoS One. 2014 Sep 3;9(9):e106447. doi: 10.1371/journal.pone.0106447 (PMC4153589; doi:10.1371/journal.pone.0106447)

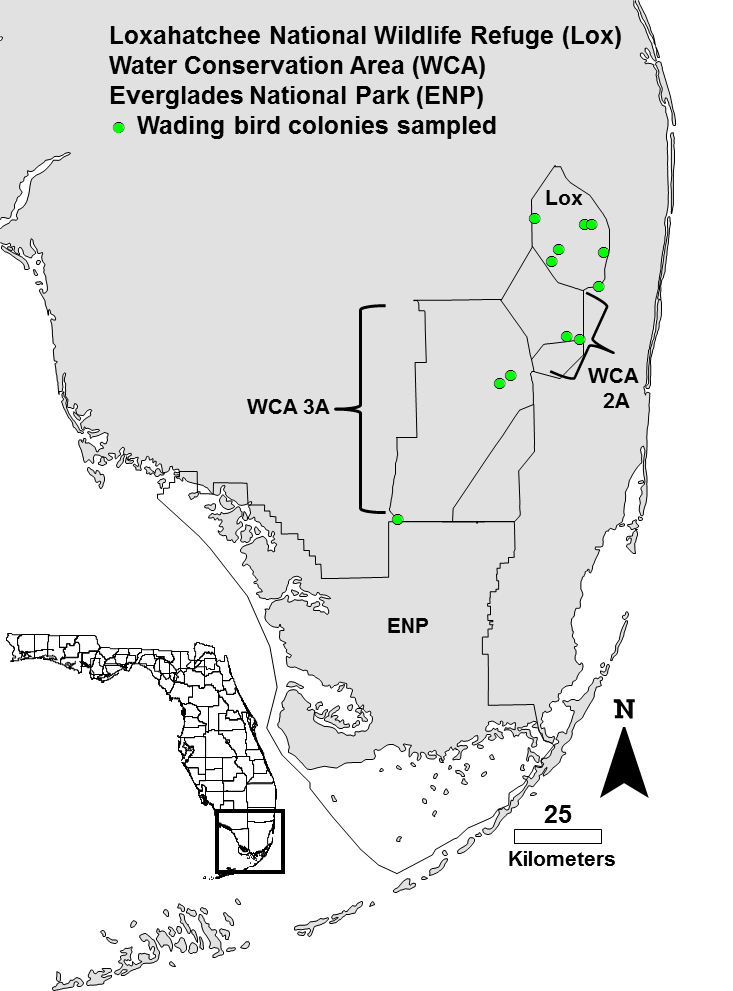

Supplement: Figure S1 — Location of study area indicating wading bird nesting colonies sampled during 2006 and 2007. The primary Water Management Areas (WCAs), Arthur R. Marshall Loxahatchee National Wildlife Refuge (Lox), and Everglades National Park (ENP) are indicated. (TIF) [file pone.0106447.s001.tif]
