# Supplementary material for: Physiological Condition of Juvenile Wading Birds in Relation to Multiple Landscape Stressors in the Florida Everglades: Effects of Hydrology, Prey Availability, and Mercury Bioaccumulation
Source: PLoS One. 2014 Sep 3;9(9):e106447. doi: 10.1371/journal.pone.0106447 (PMC4153589; doi:10.1371/journal.pone.0106447)
Supplement: Table S1 — Data used in analyses of the Physiological condition of juvenile wading birds in relation to multiple landscape stressors in the Florida Everglades: effects of hydrology, prey availability, and mercury bioaccumulation. (PDF) [file pone.0106447.s002.pdf]

Year	Species	NestID	ChickID	Hg	Hatch order	Region	Depth 2 week	Recess 2 week	Biomass 2 week	Age	HSP60	HSP70	FCORT	Mass	Tarsus
2007	GREG	gc74	gc74-1	6.33	A	WCA1	-46.62	-1.42	4.06	33	16.49		11.02	625	111
2007	GREG	gc74	gc74-2	7.63	B	WCA1	-46.62	-1.42	4.06	30	16.15		21.47	420	96
2007	GREG	gc76	gc76-1	2.41	A	WCA1	-46.62	-1.42	4.06	30	19.30	10.62	9.51	700	110
2007	GREG	gc76	gc76-2	3.01	B	WCA1	-46.62	-1.42	4.06	30	18.90	3.10		490	95
2007	GREG	gc78	gc78-1	4.52	A	WCA1	-46.62	-1.42	4.06	30	15.46	1.37	9.72	590	92
2007	GREG	gc81	gc81-1	5.59	A	WCA1	-46.62	-1.42	4.06	30	6.52	1.59	12.16	530	87
2007	GREG	gc81	gc81-2	6.44	B	WCA1	-46.62	-1.42	4.06	30	6.29	4.30		560	68
2007	GREG	gc82	gc82-1	5.54	A	WCA1	-46.62	-1.42	4.06	30	12.77	1.91	5.69	490	87
2006	GREG	gc3	gc3-1	4.91	A	WCA1	-3.74	-1.32	51.1	24	17.95		1.02	640	107.6
2006	GREG	gr216	gr216-1	6.31	A	WCA2	-3.74	-1.32	14.61	18	15.31		0.85	545	77.1
2006	GREG	gr218	gr218-1	6.04	A	WCA2	-3.74	-1.32	14.61	27	24.29	1.02	0.47	1090	123.6
2006	GREG	gc6	gc6-1	7.48	A	WCA1	-3.74	-1.32	51.1	27	7.16			705	123.8
2006	GREG	gc4	gc4-1	3.99	A	WCA1	-3.74	-1.32	51.1	30	5.47			1000	132.5
2006	GREG	gc5	gc5-1		A	WCA1	-3.74	-1.32	51.1	27	16.42		1.90	1070	153
2007	GREG	gc12	gc12-2	4.12	B	WCA1	-17.97	-1.21	12.42	9	14.64		26.72	150	36
2007	GREG	gc13	gc13-1	6.42	A	WCA1	-17.97	-1.21	12.42	15	54.24	69.87	28.81	270	49
2007	GREG	gc13	gc13-2	7.80	B	WCA1	-17.97	-1.21	12.42	15	12.62	1.03	0.84	220	45
2007	GREG	gc16	gc16-1	8.34	A	WCA1	-17.97	-1.21	12.42	9	25.90	2.56	55.74	190	41
2007	GREG	gc16	gc16-2	10.23	B	WCA1	-17.97	-1.21	12.42	9	15.38	5.07	23.49	205	42
2007	GREG	gc19	gc19-1	3.12	A	WCA1	-17.97	-1.21	12.42	12	24.55	5.56		200	40
2007	GREG	gc20	gc20-1	6.05	A	WCA1	-17.97	-1.21	12.42	9	62.08	2.49	12.80	190	33
2007	GREG	gc17	gc17-1	2.21	A	WCA1	-17.97	-1.21	12.42	15	14.75	3.98	17.14	220	48
2007	GREG	gc14	gc14-2	10.40	B	WCA1	-17.97	-1.21	12.42	9	24.74	2.47	30.78	205	38
2006	GREG	gc12	gc12-1	7.83	A	WCA1	12.65	-1.14	26.74	30			49.01	925	125.7
2007	GREG	gc42	gc42-1	1.06	A	WCA1	8.12	-0.90	0.97	12				240	40
2007	GREG	gc43	gc43-1	1.59	A	WCA1	8.12	-0.90	0.97	3			13.11	90	23
2007	GREG	gc44	gc44-1	7.96	A	WCA1	8.12	-0.90	0.97	9			9.43	160	30
2007	GREG	gc44	gc44-2	5.06	B	WCA1	8.12	-0.90	0.97	6			7.61	90	23
2007	GREG	gc45	gc45-1	0.27	A	WCA1	8.12	-0.90	0.97	12	10.18	4.00	6.95	135	33
2007	GREG	gc45	gc45-2	2.41	B	WCA1	8.12	-0.90	0.97	9			1.36	105	32
2006	GREG	gc19	gc19-1	4.98	A	WCA1	12.37	-0.75	26.74	30	11.55		0.23	760	96

2007	GREG	gc12	gc12-1	5.06	A	WCA1	-23.18	-0.70	9.68	33	7.78		11.94	805	116
2007	GREG	gc12	gc12-2	4.48	B	WCA1	-23.18	-0.70	9.68	30		23.58	11.47	630	96
2007	GREG	gc14	gc14-2	9.30	B	WCA1	-23.18	-0.70	9.68	30	10.81	2.36	21.58	580	102
2007	GREG	gc18	gc18-1	4.88	A	WCA1	-23.18	-0.70	9.68	30	6.01	2.30	11.39	360	70
2007	GREG	gc19	gc19-2	3.84	B	WCA1	-23.18	-0.70	9.68	30	22.87	3.16	11.11	550	77
2007	GREG	gc20	gc20-1	11.02	A	WCA1	-23.18	-0.70	9.68	30		5.36	7.33	570	82
2007	GREG	gc63	gc63-1	4.74	A	WCA3	13.04	-0.69	5.24	6	4.74	1.13	22.79	160	35
2007	GREG	gc67	gc67-1	6.76	A	WCA3	13.04	-0.69	5.24	12	10.22	1.95	9.19	170	33
2007	GREG	gc67	gc67-2	5.86	B	WCA3	13.04	-0.69	5.24	12	32.80	2.67	8.79	170	33
2007	GREG	gc69	gc69-2	1.61	B	WCA3	13.04	-0.69	5.24	12	7.99	2.20	6.91	170	40
2006	GREG	gr4	gr4-1	6.09	A	WCA3	1.74	-0.65	8.81	30	17.13	3.59	1.99	715	117.1
2006	GREG	gr3	gr3-1	6.47	A	WCA3	1.74	-0.65	8.81	36	0.45	0.22	0.22	890	134.7
2006	GREG	gr1	gr1-1	7.35	A	WCA3	1.74	-0.65	8.81	30	3.81	1.83	2.57	790	117.1
2006	GREG	gr2	gr2-1	5.73	A	WCA3	1.74	-0.65	8.81	36	7.98	2.66	35.19	760	122.9
2007	GREG	gc5	gc5-1	5.17	A	WCA1	-24.06	-0.47	8.06	30	2.64	3.60		655	100
2007	GREG	gc5	gc5-2	4.95	B	WCA1	-24.06	-0.47	8.06	27	14.56	2.92		390	64
2007	GREG	gc6	gc6-1	4.97	A	WCA1	-24.06	-0.47	8.06	27	7.91	4.10	5.44	645	95
2007	GREG	gc6	gc6-2	5.27	B	WCA1	-24.06	-0.47	8.06	27	9.83		3.94	550	85
2007	GREG	gc10new	gc10new-1	6.74	A	WCA1	-24.06	-0.47	8.06	21	16.61	5.12	12.93	575	80
2007	GREG	gc10new	gc10new-2	6.39	B	WCA1	-24.06	-0.47	8.06	18	12.31	16.92	9.39	560	76
2007	GREG	gc7	gc7-1	5.26	A	WCA1	-24.06	-0.47	8.06	30		37.69		480	80
2007	GREG	gc7	gc7-2	4.09	B	WCA1	-24.06	-0.47	8.06	30	25.45	31.12	4.00	550	80
2007	GREG	gc8	gc8-1	5.80	A	WCA1	-24.06	-0.47	8.06	27	32.53	18.31	9.83	705	98
2007	GREG	gc8	gc8-2	6.83	B	WCA1	-24.06	-0.47	8.06	30	58.11	5.06	3.87	570	89
2007	GREG	gc27	gc27-2	3.08	B	WCA1	-0.7	-0.43	4.99	3			2.03	105	22
2007	GREG	gc28	gc28-1		A	WCA1	-0.7	-0.43	4.99	6			3.31	160	35
2007	GREG	gc28	gc28-2	8.22	B	WCA1	-0.7	-0.43	4.99	3			27.11	75	26
2007	GREG	gc32	gc32-2	6.32	B	WCA1	-0.7	-0.43	4.99	6			11.51	149	32
2007	GREG	gc91	gc91-1	6.44	A	WCA2	-15.03	0.05	9.34	24	9.88	27.30		485	82
2007	GREG	gc91	gc91-2	9.65	B	WCA2	-15.03	0.05	9.34	21	14.85	2.77	17.66	295	71
2006	GREG	gc15	gc15-1	4.30	A	WCA1	3.44	0.11	4.88	21	19.49		0.88	530	76.4
2007	GREG	gc60	gc60-1	6.52	A	WCA3	-2.1	0.24	4.34	36	5.02	2.49	4.73	570	96
2007	GREG	gc60	gc60-2	6.12	B	WCA3	-2.1	0.24	4.34	36	24.43	9.58		575	95
2007	GREG	153.527	153.527-B	7.43	B	WCA3	1.44	0.24	4.34	39	15.65	5.26	16.21	620	89

2007	GREG	gc65	gc65-1	7.06	A	WCA3	1.44	0.27	4.34	27	12.31	1.78	6.75	565	93
2007	GREG	gc65	gc65-2	5.45	B	WCA3	1.44	0.27	4.34	27	16.84	1.44	7.74	550	85
2007	GREG	gc68	gc68-1	8.06	A	WCA3	1.44	0.27	4.34	30	18.49	1.34		585	93
2007	GREG	gc68	gc68-2	8.32	B	WCA3	1.44	0.27	4.34	27	35.25	1.55	8.10	520	80
2007	GREG	gc92	gc92-1	4.71	A	WCA2	-5.93	0.30	15.63	6			14.67	120	22
2007	GREG	gc92	gc92-2		B	WCA2	-5.93	0.30	15.63	3			6.01	105	23
2007	GREG	gc45	gc45-1	3.60	A	WCA1	-9.15	0.35	4.06	42	1.46	2.09	16.67	805	114
2007	GREG	gc44	gc44-1		A	WCA1	-9.15	0.35	4.06	39	9.83		7.76	825	120
2006	GREG	gc16	gc16-1	3.17	A	WCA3	2.66	0.48	15.15	30	35.50	0.54	0.50	755	104.5
2006	GREG	gc15	gc15-1	4.23	A	WCA1	2.66	0.48	15.15	30	26.81	3.73	0.75	730	104
2006	GREG	gc10	gc10-1	3.53	A	WCA1	2.66	0.48	15.15	21	0.51	2.95	1.03	485	70.7
2007	GREG	152.686	152.686-A	3.97	A	WCA1	-14.76	0.54	4.11	33	8.76	2.15	7.78	815	122
2007	GREG	152.686	152.686-B	3.33	B	WCA1	-14.76	0.54	4.11	30	5.27	62.77	8.11	605	90
2007	GREG	gc30	gc30-1	5.36	A	WCA1	-14.76	0.54	4.11	27	10.29		32.80	710	91
2007	GREG	gc30	gc30-2	5.44	B	WCA1	-14.76	0.54	4.11	33	5.53	1.14	34.24	445	74
2007	GREG	gc27	gc27-1	4.54	A	WCA1	-14.76	0.54	4.11	30	73.12	102.58	1.10	835	115
2007	GREG	gc27	gc27-2	5.54	B	WCA1	-14.76	0.54	4.11	27	19.68		1.47	840	120
2007	GREG	gc72	gc72-1	0.98	A	WCA1	-32.39	0.60	2.97	9		13.92		290	51
2007	GREG	gc76	gc76-1	0.39	A	WCA1	-32.39	0.60	2.97	3			14.20	70	21
2007	GREG	gc72	gc72-2	0.87	B	WCA1	-32.39	0.60	2.97	9				235	45
2006	GREG	GC2	GC2-1	5.40	A	WCA3	-26.15	0.61	14.38	24	29.94	3.39	4.27	675	93.5
2007	GREG	153.527	153.527-B	3.01	B	WCA3	10.16	0.62	5.24	18	29.18	1.42	15.69	260	47
2007	GREG	153.527	153.527-A	4.19	A	WCA3	10.16	0.62	5.24	21	10.24	1.86		475	73
2007	GREG	gc60	gc60-1	6.71	A	WCA3	10.16	0.62	5.24	15	20.50	3.97		130	33
2007	GREG	gc48	gc48-1		A	WCA3	14.88	0.65	4.66	15	11.40	0.99		265	44
2007	GREG	gc53	gc53-1	2.16	A	WCA3	14.88	0.65	4.66	15	6.46	4.14	39.83	245	47
2007	GREG	gc53	gc53-2	2.12	B	WCA3	14.88	0.65	4.66	12	6.26	2.93	23.97	160	36
2006	GREG	gc7	gc7-1	3.66	A	WCA1	12.78	0.70	22.94	21	67.00	3.69	14.01	405	61.8
2006	GREG	gc4	gc4-1	2.78	A	WCA1	16.7	0.70	22.94	33	58.83	4.20	4.20	840	125
2006	GREG	gc16	gc16-1	3.67	A	WCA1	3.44	0.70	15.15	21	2.81	0.32	0.32	500	73.9
2007	GREG	gc87	gc87-1	3.28	A	WCA2	-9.32	0.76	13.8	15	40.62	22.56	7.53	275	44
2007	GREG	gc87	gc87-2	2.70	B	WCA2	-9.32	0.76	13.8	15	52.59	7.83		280	48
2007	GREG	gc88	gc88-1	3.27	A	WCA2	-9.32	0.76	13.8	18	7.22	5.92		405	65
2007	GREG	gc88	gc88-2	3.59	B	WCA2	-9.32	0.76	13.8	18	10.08	2.67	2.44	455	69

2007	GREG	gc91	gc91-1	4.51	A	WCA2	-9.32	0.76	13.8	9	4.62	7.56	6.47	150	38
2007	GREG	gc92	gc92-1	4.09	A	WCA2	-9.32	0.76	13.8	21	18.56	3.49	1.25	675	91
2007	GREG	gc93	gc93-1	4.20	A	WCA2	-9.32	0.76	13.8	9	24.30	5.15	6.51	170	37
2007	GREG	gc94	gc94-1	2.98	A	WCA2	-9.32	0.76	13.8	15	10.52	99.10	11.83	350	62
2007	GREG	gc94	gc94-2	2.87	B	WCA2	-9.32	0.76	13.8	15	44.06	23.22	21.36	260	53
2007	GREG	gc27new	gc27new-1	3.52	A	WCA1	-3.6	0.77	7.27	12	27.21	18.15	20.20	350	49
2007	GREG	gc27new	gc27new-2	5.28	B	WCA1	-3.6	0.77	7.27	12	9.48		8.58	280	45
2007	GREG	gc27	gc27-1	4.42	A	WCA1	-3.6	0.77	7.27	15	4.91	16.65	59.03	430	65
2007	GREG	gc28	gc28-1	4.55	A	WCA1	-3.6	0.77	7.27	15	32.43	6.86	6.52	430	69
2007	GREG	gc28	gc28-2	4.97	B	WCA1	-3.6	0.77	7.27	12	8.95		10.79	320	52
2007	GREG	gc30	gc30-1	2.70	A	WCA1	-3.6	0.77	7.27	12		1.61	2.59	300	50
2007	GREG	gc30	gc30-2	2.15	B	WCA1	-3.6	0.77	7.27	12	8.83		20.67	235	42
2007	GREG	152.686	152.686-A	3.27	A	WCA1	-3.6	0.77	7.27	18	4.03	3.67	7.21	570	78
2007	GREG	152.686	152.686-B	2.21	B	WCA1	-3.6	0.77	7.27	15	22.55	12.21	15.55	250	50
2007	GREG	GC57	GC57-1	4.63	A	WCA3	17.21	0.80	3.19	3			67.89	80	25
2007	GREG	gc60	gc60-1	4.89	A	WCA3	0.3	0.81	4.04	24	25.06	48.09	4.13	385	71
2007	GREG	gc60	gc60-2	4.41	B	WCA3	0.3	0.81	4.04	24	6.71	2.65	17.78	450	65
2007	GREG	153.527	153.527-A	4.12	A	WCA3	0.3	0.81	4.04	30	8.30		8.40	670	103
2007	GREG	153.527	153.527-B	5.66	B	WCA3	0.3	0.81	4.04	30	7.44	6.29	10.56	470	68
2007	GREG	gc6	gc6-1	0.66	A	WCA1	-19.08	0.83	12.42	3			12.04	50	19
2007	GREG	gc5	gc5-1	0.58	A	WCA1	-19.08	0.83	12.42	3			15.56	115	27
2007	GREG	gc7	gc7-1	2.28	A	WCA1	-19.08	0.83	12.42	3			3.25	70	21
2006	GREG	gc17	gc17-1	5.53	A	WCA1	4.77	0.83	26.74	27	55.42		0.84	660	91.5
2006	GREG	gc18	gc18-1	5.37	A	WCA1	4.77	0.83	26.74	27	35.84		1.00	730	94.2
2006	GREG	gc20	gc20-1	6.02	A	WCA1	4.77	0.83	26.74	27	43.19	0.81	0.97	625	95.9
2007	GREG	GC63	GC63-1	4.64	A	WCA3	7.16	0.84	4.04	18	14.12	2.31	9.68	520	77
2007	GREG	gc63	gc63-2	4.25	B	WCA3	7.16	0.84	4.04	18	7.21	62.62	8.13	470	75
2007	GREG	gc65	gc65-2	2.06	B	WCA3	7.16	0.84	4.04	15	12.34	40.67	7.34	315	60
2007	GREG	gc67	gc67-1	5.19	A	WCA3	7.16	0.84	4.04	24	7.30	2.00	15.32	500	81
2007	GREG	gc67	gc67-2	5.03	B	WCA3	7.16	0.84	4.04	24	37.89	1.67	16.22	445	78
2007	GREG	gc68	gc68-1	4.58	A	WCA3	7.16	0.84	4.04	18	57.12	1.15	9.04	505	71
2007	GREG	gc68	gc68-2	4.95	B	WCA3	7.16	0.84	4.04	18	17.35	1.87	15.64	340	57
2007	GREG	gc69	gc69-2	4.71	B	WCA3	7.16	0.84	4.04	24	13.76	51.15	27.05	545	81
2007	GREG	gc48	gc48-1	2.08	A	WCA3	6.37	0.85	9.78	27	13.95	52.16	8.19	685	88

2007	GREG	gc48	gc48-2	2.47	B	WCA3	6.37	0.85	9.78	24	15.47	1.83	15.16	635	82
2007	GREG	gc53	gc53-1	1.92	A	WCA3	6.37	0.85	9.78	27	47.06		17.63	650	89
2007	GREG	gc53	gc53-2	1.82	B	WCA3	6.37	0.85	9.78	24	7.46	4.25	1.95	390	67
2007	GREG	gc43	gc43-1	2.59	A	WCA1	15.35	0.85	6.78	18	10.32	76.94	3.70	530	78
2007	GREG	gc43	gc43-2	2.35	B	WCA1	15.35	0.85	6.78	15	10.30	5.07	5.02	470	63
2007	GREG	gc44	gc44-1	2.27	A	WCA1	15.35	0.85	6.78	21	28.09	3.44	4.61	510	71
2007	GREG	gc45	gc45-1	4.83	A	WCA1	15.35	0.85	6.78	24	33.50	4.00	6.40	250	48
2006	GREG	gc17	gc17-1	5.97	A	WCA1	3.4	0.89	26.74	15	12.20		0.91	445	66.8
2006	GREG	gc18	gc18-1	6.14	A	WCA1	3.4	0.89	26.74	30	9.10		4.18	730	101.2
2007	GREG	gc49	gc49-1	3.79	A	WCA3	4	0.95	7.72	6			22.17	60	21
2006	GREG	gc12	gc12-1	4.11	A	WCA1	12.78	1.17	22.94	21	48.00	2.93	9.63	445	63
2006	GREG	gc11	gc11-1	3.35	A	WCA1	12.78	1.17	22.94	24	28.66		5.77	500	70.1
2007	GREG	gc72	gc72-1	1.79	A	WCA1	-35.72	1.34	3.83	24	7.95			660	100
2007	GREG	gc72	gc72-2	2.38	B	WCA1	-35.72	1.34	3.83	21	7.17	18.02		530	84
2007	GREG	gc74	gc74-2	3.09	B	WCA1	-35.72	1.34	3.83	18	48.52	6.94	3.92	425	73
2007	GREG	gc76	gc76-1	2.21	A	WCA1	-35.72	1.34	3.83	18	12.31	3.06	5.91	495	74
2007	GREG	gc76	gc76-2	2.17	B	WCA1	-35.72	1.34	3.83	18	3.36	33.36	8.23	425	68
2007	GREG	gc5	gc5-1	3.93	A	WCA1	-23.65	1.57	4.11	18	43.10	2.51	5.54	405	63
2007	GREG	gc7	gc7-1	5.85	A	WCA1	-23.65	1.57	4.11	15	42.72	3.51	5.92	260	46
2007	GREG	gc8	gc8-1	5.41	A	WCA1	-23.65	1.57	4.11	15	7.99	8.13	25.12	400	62
2007	GREG	gc8	gc8-2	4.81	B	WCA1	-23.65	1.57	4.11	15	8.55	3.84	8.17	290	51
2007	GREG	gc10new	gc10new-2	4.31	B	WCA1	-23.65	1.57	4.11	6	29.36	1.85	13.64	170	41
2007	GREG	gc2	gc2-1	4.08	A	WCA1	-23.65	1.57	4.11	9	7.80	0.91		480	83
2007	GREG	gc2	gc2-2		B	WCA1	-23.65	1.57	4.11	9	4.50	1.53	5.12	470	80
2007	GREG	gc6	gc6-1	6.55	A	WCA1	-23.65	1.57	4.11	15	34.58	1.31	27.57	300	56
2007	GREG	gc6	gc6-2	3.82	B	WCA1	-23.65	1.57	4.11	15		20.08	7.18	300	59
2006	GREG	gc10	gc10-1	0.88	A	WCA1	3.44	1.89	15.15	12	7.86		1.94	365	57.2
2007	GREG	gc13	gc13-1	6.55	A	WCA1	-23.26	1.93	8.16	24	18.23	1.40	3.04	620	87
2007	GREG	gc13	gc13-2	6.85	B	WCA1	-23.26	1.93	8.16	24	9.06	2.28		510	73
2007	GREG	gc14	gc14-1	9.06	A	WCA1	-23.26	1.93	8.16	18	15.73	2.20		590	84
2007	GREG	gc14	gc14-2	9.46	B	WCA1	-23.26	1.93	8.16	18	24.75	13.62		455	72
2007	GREG	gc15	gc15-1	5.42	A	WCA1	-23.26	1.93	8.16	18	32.74	16.86	3.22	520	75
2007	GREG	gC15	gC15-2	5.77	B	WCA1	-23.26	1.93	8.16	18	10.49	2.27	23.60	355	63
2007	GREG	gc16	gc16-1	9.17	A	WCA1	-23.26	1.93	8.16	18	3.31	1.62	8.80	420	73

2007	GREG	gc16	gc16-2	9.21	B	WCA1	-23.26	1.93	8.16	18	7.14	2.90	11.94	410	68
2007	GREG	gc17	gc17-1	3.10	A	WCA1	-23.26	1.93	8.16	24	7.23	3.79	11.54	350	64
2007	GREG	gc18	gc18-1	3.29	A	WCA1	-23.26	1.93	8.16	18	28.37	8.25	3.06	280	43
2007	GREG	gc20	gc20-1	7.33	A	WCA1	-23.26	1.93	8.16	18	30.89	2.33	7.83	400	60
2007	GREG	gc20	gc20-2	5.96	B	WCA1	-23.26	1.93	8.16	15	6.00	8.79	16.48	330	55
2007	GREG	gc12	gc12-2	5.11	B	WCA1	-23.26	1.93	8.16	18	33.67	3.20	11.73	380	70
2006	WHIB	wc364	wc364-1	0.12	A	WCA1	13.74	-2.30	15.26	18	68.28		0.18	545	71.7
2006	WHIB	wc363	wc363-1	0.10	A	WCA1	13.74	-2.30	15.26	18	45.08	1.91	16.33	505	69.8
2006	WHIB	wc362	wc362-1	0.56	A	WCA1	13.74	-2.30	15.26	18	0.98		12.82	690	84.2
2006	WHIB	wcr100	wcr100-1	0.26	A	WCA1	13.74	-2.30	15.26	18	24.31	3.10	0.93	435	63.5
2007	WHIB	wc72	wc72-1	2.49	A	WCA1	-33.77	-0.76	6.78	3			12.69	75	20
2007	WHIB	wc62	wc62-1	0.24	A	WCA1	-33.77	-0.76	6.78	6			12.61	190	37
2007	WHIB	wc22	wc22-2	0.30	B	WCA1	9.73	-0.76	6.78	6			10.26	140	31
2007	WHIB	wc22	wc22-1	0.04	A	WCA1	9.73	-0.76	6.78	9			17.98	290	45
2006	WHIB	wcr163	wcr163-1	0.17	A	WCA3	-26.37	-0.65	8.81	27	13.15		0.04	520	79.6
2006	WHIB	wc3	wc3-1	0.18	A	WCA1	1.59	-0.23	1.83	24	18.07		14.61	605	80.2
2006	WHIB	wc76	wc76-2	0.56	B	WCA1	9.35	-0.14	26.74	12	29.46		0.01	292	42.1
2006	WHIB	wv72	wv72-1	0.23	A	WCA1	9.35	-0.14	26.74	15	37.82		24.61	304	46.2
2006	WHIB	wc79	wc79-2	0.23	B	WCA1	9.35	-0.14	26.74	12	20.33		2.00	298	43.8
2006	WHIB	91316	91316-1	0.18	A	WCA1	9.35	-0.14	26.74	6	29.18	2.72	3.49	153	30.6
2006	WHIB	wc72	wc72-2	0.28	B	WCA1	9.35	-0.14	26.74	12	2.85	3.80	8.33	300	44.7
2006	WHIB	wc76	wc76-1	0.73	A	WCA1	9.35	-0.14	26.74	15	7.79		2.49	295	45.9
2006	WHIB	91320	91320-1	0.37	A	WCA1	9.35	-0.14	26.74	6	8.49	1.99	3.20	154	30.1
2006	WHIB	91304	91304-1	0.55	A	WCA1	9.35	-0.14	26.74	6	9.99		1.36	134	28.7
2006	WHIB	91321	91321-1	0.35	A	WCA1	9.35	-0.14	26.74	6	50.49	2.76	0.04	127	26.2
2007	WHIB	wc77	wc77-1	0.72	A	WCA1	-13.16	0.41	4.06	24	18.62	3.19	20.91	385	62
2007	WHIB	hid13	hid13-1	0.34	A	WCA3	-2.5	0.57	4.65	9	6.77	1.66	20.94	215	43
2007	WHIB	wc1	wc1-1	6.58	A	WCA1	-2.3	0.62	6.78	6	35.03	2.62		370	48
2007	WHIB	wc2	wc2-2	2.21	B	WCA1	-2.3	0.62	6.78	3				110	26
2007	WHIB	wc10	wc10-1	1.23	A	WCA1	-2.3	0.62	6.78	6				150	29
2007	WHIB	wc10	wc10-2	1.53	B	WCA1	-2.3	0.62	6.78	3				70	21
2007	WHIB	wc11	wc11-1	0.92	A	WCA1	-2.3	0.62	6.78	3				70	21
2007	WHIB	wc12	wc12-2	0.40	B	WCA1	-2.3	0.62	6.78	6				160	29
2007	WHIB	wc17	wc17-2	8.19	B	WCA1	-2.3	0.62	6.78	3				110	25

2007	WHIB	wc2	wc2-1	0.30	A	WCA1	-2.3	0.62	6.78	6	19.96	1.63		175	33
2007	WHIB	wc14	wc14-1	0.28	A	WCA1	3.38	0.62	6.78	3		1.88		110	22
2007	WHIB	wc42	wc42-1	0.13	A	WCA1	-3.59	0.63	7.52	15	4.13	16.58	18.42	495	70
2007	WHIB	wc44	wc44-1	0.17	A	WCA1	-3.59	0.63	7.52	12	10.82	47.84	7.59	420	61
2007	WHIB	wc49	wc49-1	0.38	A	WCA1	-3.59	0.63	7.52	9	66.28	1.57	26.74	260	45
2007	WHIB	wc50	wc50-1	0.16	A	WCA1	-3.59	0.63	7.52	12	66.64	1.10	15.19	495	63
2007	WHIB	wc51	wc51-1	0.39	A	WCA1	-3.59	0.63	7.52	12	35.01	0.83	20.63	310	50
2007	WHIB	wc76	wc76-1	0.13	A	WCA1	0.16	0.63	7.52	6	29.16	2.97	5.80	205	34
2006	WHIB	wc10	wc10-1	3.67	A	WCA3	-26.37	0.70	14.38	18	1.60	1.29	0.37	275	57.2
2006	WHIB	WC6	WC6-1	0.85	A	WCA3	-26.37	0.70	14.38	15	2.14	6.46	0.92	290	44.3
2006	WHIB	44190	44190-1	6.62	A	WCA1	9.73	0.75	26.74	3	10.85		4.02	80	24
2007	WHIB	wrc	wrc-1	1.97	A	WCA3	-4.37	0.76	11.55	15	5.45	1.31	7.20	320	53
2007	WHIB	wrc	wrc-2	1.45	A	WCA3	-4.37	0.76	11.55	15	49.39	1.01	5.49	340	55
2007	WHIB	wrc	wrc-3	0.39	A	WCA3	-4.37	0.76	11.55	15	12.91	0.94	7.04	200	45
2007	WHIB	wrc	wrc-5	1.47	A	WCA3	-4.37	0.76	11.55	15	16.19	1.26	14.70	315	60
2007	WHIB	wrc	wrc-6	0.61	A	WCA3	-4.37	0.76	11.55	21	8.09	2.26	34.40	570	79
2007	WHIB	wc64	wc64-1	0.32	A	WCA1	-42.51	0.83	6.78	18	9.98	1.13	6.63	350	53
2007	WHIB	wc68	wc68-1	0.88	A	WCA1	-42.51	0.83	6.78	18	16.48	1.41	7.43	465	69
2007	WHIB	wr11	wr11	0.18	A	WCA1	9.73	0.85	6.78	24	2.49	0.89	6.25	540	66
2007	WHIB	wr1	wr1	0.31	A	WCA1	9.73	0.85	6.78	24	35.13	71.29		420	66
2007	WHIB	wr2	wr2	0.27	A	WCA1	9.73	0.85	6.78	21	28.22	1.60	8.05	345	58
2007	WHIB	wr3	wr3	0.22	A	WCA1	9.73	0.85	6.78	26	18.22	1.73		390	66
2007	WHIB	wr4	wr4	0.05	A	WCA1	9.73	0.85	6.78	30	5.34	2.55	8.80	680	82
2007	WHIB	wr5	wr5	0.37	A	WCA1	9.73	0.85	6.78	24	20.63	1.61		360	59
2007	WHIB	wr6	wr6	0.49	A	WCA1	9.73	0.85	6.78	24	10.04		42.82	500	72
2007	WHIB	wr8	wr8	0.11	A	WCA1	9.73	0.85	6.78	24	17.10	3.80	8.16	480	75
2007	WHIB	wr9	wr9	0.09	A	WCA1	9.73	0.85	6.78	24	7.43	1.31	12.99	510	70
2007	WHIB	wcsg	wcsg-1	0.42	A	WCA1	9.73	0.85	6.78	12	10.24	3.18	7.11	360	56
2006	WHIB	91316	91316-1	0.20	A	WCA1	9.73	0.97	15.26	12	42.95	2.47	4.89	317	44.2
2006	WHIB	91330	91330-1	0.62	A	WCA1	9.73	0.97	15.26	12	8.55	4.72	6.22	308	43.1
2006	WHIB	91346	91346-1	0.81	A	WCA1	9.73	0.97	15.26	12	27.33		4.14	321	44.2
2006	WHIB	91311	91311-1	0.54	A	WCA1	9.73	0.97	15.26	12	27.41		1.36	310	44.7
2006	WHIB	91319	91319-1	0.57	A	WCA1	9.73	0.97	15.26	12		5.58	1.15	340	51
2006	WHIB	91309	91309-1	0.39	A	WCA1	9.73	0.97	15.26	12		4.43	4.43	340	50

2007	WHIB	wc34	wc34-1	1.68	A	WCA3	7.68	1.15	3.57	3			12.77	120	31
2007	WHIB	wc34	wc34-2	4.36	B	WCA3	7.68	1.15	3.57	3			2.98	85	26
2007	WHIB	wc35	wc35-1	3.12	A	WCA3	7.68	1.15	3.57	6			11.43	170	35
2007	WHIB	wc35	wc35-2	1.73	B	WCA3	7.68	1.15	3.57	3				100	29
2007	WHIB	wc36	wc36-1	2.07	A	WCA3	7.68	1.15	3.57	3			10.64	100	29
2007	WHIB	wc36	wc36-2	1.23	B	WCA3	7.68	1.15	3.57	3			6.77	95	30
2007	WHIB	wc37	wc37-2	3.16	B	WCA3	7.68	1.15	3.57	3			3.62	80	25
2007	WHIB	wc38	wc38-2	1.50	B	WCA3	7.68	1.15	3.57	3			15.58	60	20
2007	WHIB	wc40	wc40-1	2.53	A	WCA3	7.68	1.15	3.57	6			7.09	190	40
2007	WHIB	wc27	wc27-1	0.36	A	WCA3	7.68	1.15	3.57	6			3.53	135	30
2007	WHIB	wc27	wc27-2	1.62	B	WCA3	7.68	1.15	3.57	3			11.01	80	24
2007	WHIB	wc28	wc28-2	0.57	B	WCA3	7.68	1.15	3.57	3			4.01	115	25
2007	WHIB	wc29	wc29-1	0.67	A	WCA3	7.68	1.15	3.57	9	16.85		3.79	260	40
2007	WHIB	wc31	wc31-1	2.31	A	WCA3	7.68	1.15	3.57	6			3.44	230	36
2007	WHIB	wc30	wc30-1	2.12	A	WCA3	7.68	1.15	3.57	6				195	32
2007	WHIB	wc30	wc30-2	3.13	B	WCA3	7.68	1.15	3.57	3			22.09	120	29
2006	WHIB	91319	91319-1	0.99	A	WCA1	9.73	1.39	15.26	21	24.74		0.65	570.4	66.7
2006	WHIB	91306	91306-1	0.17	A	WCA1	9.73	1.39	15.26	21	40.82	1.08	3.61	570	67
2006	WHIB	91304	91304-1	0.26	A	WCA1	9.73	1.39	15.26	21	8.20		8.30	550	66
2006	WHIB	91333	91333-1	0.41	A	WCA1	9.35	1.59	15.15	6	35.76		0.25	120	26.8
2006	WHIB	wcr151	wcr151-4	2.11	A	WCA1	10.45	1.59	15.15	18	1.16	2.81	4.12	350	54.4
